# Editorial: Genitourinary (GU) oncology in low-to-middle income countries

**DOI:** 10.3389/fruro.2023.1172370

**Published:** 2023-03-30

**Authors:** Mohammed Shahait, Muhannad Alsyouf, Ibrahim Abu-Gheida, Fernando Maluf

**Affiliations:** ^1^ Department of Surgery, King Hussein Medical Center, Amman, Jordan; ^2^ Department of Urology, University of Sharjah, Sharjah, United Arab Emirates; ^3^ Department of Urology, University of Southern California, Los Angeles, CA, United States; ^4^ Department of Radiation Oncology, Cleveland Clinic Abu Dhabi, Abu Dhabi, United Arab Emirates; ^5^ Department of Medicine, Albert Einstein Israelite Hospital, São Paulo, Brazil

**Keywords:** low to middle income countries, oncology, disparity, access and quality, urology

While cancer is accountable for one in every six deaths around the world, 70% of those deaths are registered in low-middle income countries (LMICs) (1). As such, Genitourinary (GU) malignancies such as prostate, renal, bladder, urethral, testicular, and penile cancers represent almost 12.7% of all newly diagnosed cancer in the world in 2020 and 8% of cancer death globally (2).

Over the last few decades, several local and international initiatives succeeded in improving resources allocations to curb cancer associated mortality and to mitigate its impact on quality of life; nevertheless, only 5% of these resources are designated for LMICs (3).

There is scarce data available in regards to the nature and the challenges of genitourinary (GU) oncology practice in LMICs across the globe. Therefore, there is an exigent need to address the status of GU oncology practice in this part of the world. In this special issue of Frontiers urology, we aimed to provide insight about different aspects of GU oncology practice and research in LMICs.

In this Research Topic’s first article, Ibrahim et al. from the Global Health Institute at the American University of Beirut, conducted a bibliometric analysis of scientific publications on GU cancer in 22 Arab countries. This type of research helps in understanding the growth, impact, trends, and flow of knowledge within GU cancer research. The authors found that despite increasing research productivity in the Arab World; the quality, focus topics, and cross-country collaborations are still lagging, compounded by under-representation of women in GU cancer research. The findings from this study act as a harbinger bell for building research infrastructure, capacity, and collaborations.

Likewise, Choksi et al. assessed disparities in clinical trials for benign and malignant urological pathologies in low to middle income countries. Across the different search queries, LMICs represented a higher proportion of non-oncologic urologic clinical trials as compared to their representation of urologic oncology clinical trials (z = -16.05, p < 0.001). For example, only 1.83% of prostate cancer clinical trials registered in two databases are being performed in LMICs. On the other hand, clinical trials to address the management of lower urinary tract symptoms in LMICs were 8.33%. Furthermore, the authors found a weak relationship between the global burden of disease and the number of clinical trials conducted in each country for different urologic conditions.

In developed countries, access to new systemic treatments for different GU malignancies is influenced by several factors such as race, socio-economic status, and the structure of healthcare. However, in LMIC, patients face more intricate challenges to receive novel systematic therapies for GU malignancies. This was eloquently described by Herchenhorn et al. in their comprehensive review in which they proposed a multifaceted framework to improve access to new systematic treatments in LMICs, improve medications pricing, and consideration in future clinical trials design to mitigate the cost.

The “Obesity paradox” is a well-studied phenomenon in oncology field, where obese patients tend to have improved overall survival compared to normal weight patients, yet data replicating these findings in countries outside of North America and Europe are sparse (Moreira et al.). Moreira et al. performed a retrospective, multicenter study to assess the clinical outcome including overall survival (OS) of Brazilian patients with advanced cancer treated with ICI according to baseline body mass index (BMI). This cohort composed predominantly of patients with stage IV lung cancer (57%) and melanoma (19%). There was no statistically significant difference in the median OS of the obese group to the non-obese group (BMI < 30 kg/m2).These findings emphasize that observations made in cohorts from clinical trials conducted in developed countries might not be perceived in real-world setting.

The disparities between countries in the treatment of GU malignancies is evident, and more pronounced in LMICs. This gap in care is exacerbated by the shortage of physicians in countries with insufficient resources and exodus of physicians to work in higher income countries. Also, lack of funding, inability to negotiate drug pricing, and lack of education and training opportunities for healthcare professionals play a major role in the disparity observed in the treatment of different GU malignancies. We also believe that the World Health Organization (WHO), and other international bodies, should develop comprehensive programs to tackle the different issues facing healthcare systems in LMICs.

## Author contributions

MS: Writing. MA: Writing and editing. IG: Editing. FM: Writing, editing, and supervision. All authors contributed to the article and approved the submitted version.
